# A SU6668 pure nanoparticle-based eyedrops: toward its high drug Accumulation and Long-time treatment for corneal neovascularization

**DOI:** 10.1186/s12951-024-02510-8

**Published:** 2024-05-27

**Authors:** Han Wu, Jinfa Ye, Minjie Zhang, Lingyu Zhang, Sijie Lin, Qingjian Li, Yanbo Liu, Yun Han, Caihong Huang, Yiming Wu, Yuhang Cheng, Shundong Cai, Lang Ke, Gang Liu, Wei Li, Chengchao Chu

**Affiliations:** 1 School of Medicine, Xiamen University Affiliated Xiamen Eye Center, Eye Institute of Xiamen University, Xiamen University, Xiamen, 361102 China; 2 Fujian Provincial Key Laboratory of Ophthalmology and Visual Science, Fujian Engineering and Research Center of Eye Regenerative Medicine, Xiamen, 361102 China; 3 State Key Laboratory of Physical Chemistry of Solid Surfaces, The MOE Key Laboratory of Spectrochemical Analysis & Instrumentation, College of Chemistry and Chemical Engineering, Xiamen University, Xiamen, 361002 China; 4 Department of Rheumatology and Clinical Immunology, School of Medicine, the First Affiliated Hospital of Xiamen University, Xiamen University, Xiamen, XM 361000 China; 5 Municipal Clinical Research Center for Immune Diseases, Xiamen, XM 361000 China; 6 Xiamen Key Laboratory of Rheumatology and Clinical Immunology, Xiamen, XM 361000 China; 7 Shen Zhen Research Institute of Xiamen University, Shenzhen, 518057 China

**Keywords:** Corneal neovascularization, Pure drug nanoparticle, SU6668, Cell membrane vesicle

## Abstract

Corneal neovascularization (CNV) is one of the common blinding factors worldwide, leading to reduced vision or even blindness. However, current treatments such as surgical intervention and anti-VEGF agent therapy still have some shortcomings or evoke some adverse effects. Recently, SU6668, an inhibitor targeting angiogenic tyrosine kinases, has demonstrated growth inhibition of neovascularization. But the hydrophobicity and low ocular bioavailability limit its application in cornea. Hereby, we proposed the preparation of SU6668 pure nanoparticles (NanoSU6668; size ~135 nm) using a super-stable pure-nanomedicine formulation technology (SPFT), which possessed uniform particle size and excellent aqueous dispersion at 1 mg/mL. Furthermore, mesenchymal stem cell membrane vesicle (MSCm) was coated on the surface of NanoSU6668, and then conjugated with TAT cell penetrating peptide, preparing multifunctional TAT-MSCm@NanoSU6668 (T-MNS). The T-MNS at a concentration of 200 µg/mL was treated for CNV *via* eye drops, and accumulated in blood vessels with a high targeting performance, resulting in elimination of blood vessels and recovery of cornea transparency after 4 days of treatment. Meanwhile, drug safety test confirmed that T-MNS did not cause any damage to cornea, retina and other eye tissues. In conclusion, the T-MNS eye drop had the potential to treat CNV effectively and safely in a low dosing frequency, which broke new ground for CNV theranostics.

**Supplementary Information:**

The online version contains supplementary material available at 10.1186/s12951-024-02510-8.

## Introduction

The cornea is transparent and free of blood vessels and lymph. This avascular state is maintained by low levels of pro-angiogenic factors and high levels of anti-angiogenic factors [1]. However, various pathological conditions such as corneal infection, severe alkali burns, corneal limbal stem cell deficiency, ocular trauma, allergies, and other ocular surface diseases can disturb the balance between angiogenic and anti-angiogenic factors, ultimately resulting in the development of corneal neovascularization (CNV) [2, 3]. Nowadays, CNV is one of the common blinding factors worldwide which leads to reduced vision or even blindness. However, conventional surgical interventions may trigger an inflammatory response, further exacerbating CNV [4]. Meanwhile, advanced photocoagulation techniques requiring high laser energy can induce various complications, including blood vessel occlusion, corneal hemorrhage, corneal thinning or hyperthermic injuries [5]. Anti-vascular endothelial growth factor (VEGF) agent therapy such as bevacizumab, ranibizumab, pegaptanib and aflibercept have demonstrated efficacy in inhibiting CNV [6–8]. But the conventional route of anti-VEGF drugs for CNV treatment has the disadvantages of repeated injections, low corneal penetration, high drug clearance, short duration of action and drug resistance, resulting in poor efficacy [9, 10]. This motivates the researchers to develop new anti-angiogenic formulations for the treatment of CNV.

It has been demonstrated that VEGFR2 plays a crucial role in mediating VEGF-induced neovascularization, VEGF/VEGFR system is the main angiogenic modulator and it is closely related to the MMPs family, such as MMP2 and MMP9 [11–13]. Among anti-neovascularization drugs, SU6668, a small molecule inhibitor of the tyrosine kinase activity of three angiogenic receptors VEGFR2, PDGFRβ and FGFR1, has been considered a potent substance for antagonizing central pathways of angiogenic signal transduction [14, 15]. Up to now, SU6668 has shown promising antiangiogenic effects on primary tumors [14], and its safety and non-toxicity have been confirmed through Phase I clinical trials [16, 17]. While there are no reports of SU6668 being used for CNV treatment, it holds potential as a drug for CNV treatment due to its unique properties. However, its hydrophobic property limits the further application as an eyedrops.

In recent decades, nanoparticles have been widely investigated as an innovative drug delivery platform for CNV [18–20]. Various nano-delivery systems, such as micelle or polymer, can not only improve bioavailability, water solubility and penetration of drugs into cornea, but also prolong drug retention time, thereby achieving sustained delivery and controlled therapeutic release with minimal toxicity and side effects [21–24]. In our previous studies, we introduced a super-stable pure-nanomedicine formulation technology (SPFT) using a supercritical fluid antisolvent strategy. This approach allows for the preparation of pure drug nanoparticles without the addition of other delivery vehicles. Moreover, our strategy shows potential in enhancing the water solubility of hydrophobic drugs, improving drug delivery efficiency, and extending the circulation time of drugs in the bloodstream.

In this study, the SU6668 pure nanoparticles (NanoSU6668) were synthesized using the SPFT strategy, and the obtained NanoSU6668 exhibited spherical nanostructures with a uniform particle size and excellent aqueous dispersion. In recent years, cell membrane vesicles (MVs) have been applied to encapsulate on the surface of nanomedicine, resulting in prolonged blood circulation time, enhanced biocompatibility, and increased drug bioavailability [25–27]. Building upon this knowledge base, NanoSU6668 was encapsulated by mesenchymal stem cells (MSCs) derived MVs [28–31]. Subsequently, the surface of the encapsulated NanoSU6668 was modified with TAT peptide. The intact MSC membrane surface proteins maintained the targeting neovascularization properties, while the TAT peptide promoted tissue permeability [32]. The prepared TAT-MSCm@NanoSU6668 (T-MNS) was applied in CNV treatment. Unlike previous studies, our research takes into account various characteristics of CNV drug delivery, including material composition, concentration, biodistribution, size, shape, biocompatibility, surface charge and zeta potential. The modified drug T-MNS avoids nanotoxicity and achieves improved drug targeting and accumulation in CNV treatment [21, 33]. Consequently, T-MNS was administered to the CNV model *via* eye drops, and demonstrated outstanding safety and therapeutic effects.

## Materials and methods

### Preparation and characterization of NanoSU6668

Firstly, 100 mg SU6668 (T125079, Aladdin, Shanghai, China) was dissolved in 10 mL mixed solution of dimethyl sulfoxide (DMSO, D8370, Solarbio, Beijing, China) and ethanol (E111989, Aladdin), the solution pump to have reached a supercritical state of supercritical carbon dioxide (SC - CO_2_) reactor. While maintaining the supercritical state, CO_2_ was pumped continuously at a certain rate to remove the mixed solution, resulting in the precipitation of SU6668. Continuous passage of CO_2_ was carried out until NanoSU6668 was dried, resulting in the obtainment of NanoSU6668. Besides, 100 mg SU6668 and 5 mg indocyanine green (ICG) were dissolved in 10 mL mixed solution of DMSO and ethanol, following the same method as above, resulting in the precipitation of NanoSU6668 (ICG). Continuous passage of CO_2_ was maintained until NanoSU6668 (ICG) was dried, leading to the obtainment of NanoSU6668 (ICG). The morphology of the nanoparticles was studied by transmission electron microscope (TEM) (model 1200 EXII; JEOL, Tokyo, Japan), while the size distribution and zeta potential of Nanoparticles (NPs) were measured using Zetasizer (Malvern, UK).

### Preparation and characterization of TAT-MSCm@NanoSU6668

We isolated MSCs from bone marrow of rat femurs [34, 35]. Briefly, 28-day-old male Sprague Dawley rats were obtained from Shanghai SLAC laboratory Animal Co., Ltd. And, all the rats were raised at Animal Care and Use Committee of Xiamen University (Ethics number XMULAC20190146). Bone femurs were aseptically dissected from rats and collected on ice in isolation medium, each femur was flushed with complete culture medium (CCM) and bone marrow cells were collected. Then, the cells were cultured in a T-75 tissue culture flasks after filtered through a 70 μm nylon cell strainer. Cells were incubated at 37 °C under 5% CO2, and nonadherent cells were removed by change of medium after 24 h. After 3 days, cells were retrieved and counted. In order to isolate immature MSCs, cells were seeded in serum-free CCM into fibronectin-coated plates, following 20 min incubation, nonadherent cells were washed away with PBS. The adherent cells were then cultured in CCM, the culture medium was changed every 2–3 days. When the cell density reached 80% confluence, flow cytometry analysis for CD90 cell surface marker was performed, confirming that the isolated cells were MSCs, as CD90 is the accepted standard marker of MSCs [36]. Briefly, 1.8 µL APC anti-rat CD90/mouse CD90.1 (202,526, Biolegend, USA) was added to a 96-well V-bottom plate; 100 µL of MACS (Miltenyi Biotec, USA) buffer and 100 µL cell suspension were added to each well containing an antibody. The plate was incubated at 4 °C for 30 min, shielded from light, and afterward was centrifuged for 5 min at 300 g, cells were washed and resuspended in 200 µL of MACS buffer. A Cytoflex LX flow cytometer (Beckman Coulter, USA) was used for data collection, and data were analyzed using FlowJo V10 software (FlowJo, USA). Then the MVs were prepared according to our previous study [27]. Firstly, the cells were lysed on a shaker at 4 ℃ for 5 h, followed by sonication with an ultrasonic probe at 20% amplitude for 3 cycles, each lasting 20 s. The cell lysate was centrifuged at 500 g for 3 times, each lasting 10 min, and then the supernatant was centrifuged for 30 min at 10,000 g. The microspheres were suspended in PBS after the supernatant was further centrifuged at 70,000 g for 90 min, the above centrifugation processes were carried out at 4 ℃. The membrane protein concentrations of MVs were determined using the BCA Protein Assay kit (Vazyme, Nanjing, China) and stored at − 80 °C for following researches.

Afterwards, the MVs was mixed with NanoSU6668 or NanoSU6668 (ICG) and extruded with a liposome extruder to prepare multifunctional bionic drugs MSCm@NanoSU6668 (MNS) or MNS (ICG) (Avanti Polar Lipids).

In the further study, TAT peptide (RKKRRQRRR) with an N-Hydroxysuccinimide (NHS) active ester to the C-terminus (TAT-NHS) was purchased from China Peptides Co., Ltd. Under the condition of physiological TAT-NHS could anchored on the surface of the cell membrane. In brief, 1 mg/mL TAT-NHS (based on TAT concentration), mixed thoroughly with 2 mg/mL MSCm (based on membrane protein), was reacted at 4 °C for 4 h, and then dialyzed in a 3.5 kDa semi-permeable membrane to remove unreacted TAT-NHS. To confirm the conjugation of TAT peptide with MVs, we mixed MSCm with FITC-modified TAT-NHS, and then examined the vesicles using confocal microscopy (Zeiss LSM 880 + Airyscan, Germany) after the reaction. Finally, TAT-MSCm@NanoSU6668 (T-MNS) or T-MNS (ICG) were prepared.

### Cell viability assay

The cytotoxicity of MSCm, NanoSU6668, MNS, and T-MNS were determined using the Cell Counting Assay Kit-8 assay (40203ES76, Yeasen, Shanghai, China). In brief, human umbilical vein endothelial cells (HUVECs) were seeded into 96-well culture plates (1 × 10^4^ HUVECs/well) for 12 h, then the cells were changed to fresh medium containing drugs with different concentrations (6.25, 12.5, 25, 50 µM). After cultured 24 h, the cells were washed with PBS and detected with CCK8 kits. The absorbance at 450 nm was measured using a microplate reader (Bio-Tek, Winooski, VT). In addition, the cytotoxicity of MSCm, NanoSU6668, MNS, and T-MNS to the primary rat corneal endothelial cells (CECs) and human corneal epithelial cells (HCEs) were also tested.

### Fluorescent image studies

Cellular uptake of the drug delivery system was studied by fluorescent image studies. HUVECs, CECs, and HCEs were cultured on glass-bottomed culture dishes at a density of 5 × 10^5^ cells/dish for 12 h. Subsequently, NanoSU6668 (ICG), MNS (ICG) and T-MNS (ICG) (25 µM) were added to the culture medium and incubated for an additional 6 h. Following a wash with PBS, all cells were stained with 4′,6-diamidino-2-phenylindole (DAPI). Finally, the cells were fixed with 4% Paraformaldehyde, and fluorescence images of the cells were investigated using a confocal fluorescence microscope (Zeiss LSM 880 + Airyscan, Germany).

### Migration ability assay

A scratch test was used to determine changes in the migratory ability of HUVECs after different treatments. Firstly, HUVECs were cultured in a twelve-well plate, and a scratch model was created by drawing a straight line on the lower chamber surface using a 200-µL pipette tip, Subsequently, PBS was utilized to wash off any exfoliated cells. Afterwards, fresh media containing MSCm, NanoSU6668, MNS and T-MNS (25 µM) was added to the plates, and cells were incubated continuously for 12 h. The images were obtained by a fluorescent inverted microscope (Nikon Ti-U), and the relative migration rate was calculated by Image J.

### Tube formation assay

The endothelial tube formation was a crucial criterion in assessing the efficacy of anti-angiogenesis treatments, thus the tube formation assay was investigated. Firstly, HUVECs were treated with MSCm, NanoSU6668, MNS, and T-MNS (25 µM) for 12 h. Simultaneously, a 96-well plate was pre-cooled to − 20 °C prior to the experiments. Subsequently, Matrigel (354,277, Corning, USA) was laid on the bottom of the plates and allowed to solidify at 37 °C for 2 h. Secondly, the cells were collected and seeded on top of the Matrigel to each well. After cultured 8 h, the tube formation was investigated by a fluorescent inverted microscope (Nikon Ti-U), and the number of junction points were calculated by Image J.

### CNV model establishment

Once the rats reached a weight of 200 g, the CNV models were constructed on their left eyes by central corneal suture technique [37]. After 10 days, new blood vessels were found obviously in rat corneas under the slit lamp microscope, and the CNV images were captured using an Image Pro Plus version 6.0 (Media Cybernetics, Silver Spring, MD, USA). The neovascularization network was evenly distributed at trans-circular clock points and coarse meandering neovascularization growing toward the center of the cornea was considered an ideal CNV model for our experiment [37, 38].

### Treatment and evaluation of CNV in vivo

Following the construction of the CNV animal model, the rats were divided into two treatment groups: Group A (*n* = 6), which included PBS, MSCm, NanoSU6668 (ICG), MNS (ICG), and T-MNS (ICG), Group B (*n* = 6), which included PBS, MSCm, NanoSU6668, MNS, and T-MNS. The concentration of all drugs in Group A and Group B were maintained at 200 µg/mL. The drugs were administered topically to neovascular eyes *via* eye drops at a dosage of 10 µL.

To trace the drug accumulation in CNV, in vivo PA oxygen saturation imaging (PAI-o.s.) and PAI-ICG of the neovascularized corneas in Group A were obtained with a Vevo 3100 (VisualSonics FujiFilm) at 3, 6, 12, and 24 h time points after the first treatment. Following the in vivo test, corneal specimens from the T-MNS (ICG) treatment group were promptly excised and fixed subsequent to euthanization. One-micrometer sections were stained with methylene blue and corneal tissues were then thin sectioned on an ultramicrotome (LKB, Gaithersburg, MD, USA). Sections were collected on 150-mesh grids, stained with uranyl acetate and lead citrate, examined, and photographed using a transmission electron microscope (model 1200 EXII; JEOL, Tokyo, Japan).

In order to investigate the therapeutic efficacy of the drugs, the administration for the Group B was conducted twice daily (at 9:00 and 21:00) for a duration of 4 days. During this treatment period, the corneas of all the rats were observed under slit lamp microscope every day. To quantify the therapeutic effect of each drug on CNV, the length of the longest neovascularization growing vertically from the corneal rim in four quadrants was measured and calculated according to the CNV area formula: S = C/12 × 3.1416[r^2^−(r − l)^2^], where C is the number of trans-circular clock points of the neovascular network, r is the radius of the rat cornea, l is the length of the neovascularization with small continuous curvature perpendicular to a line tangential to the cornea, and the average of the maximum vessel length in the four quadrants was calculated [37, 38]. Additionally, the corneal stromal neovascularization of the treated rats was examined with a laser-scanning in vivo confocal microscope with a corneal imaging module (HRT3-RCM, Heidelberg Engineering, Germany) when the 4 days treatment was completed.

At the end of treatments, all rats of Group B were executed and the eyeballs were excised. Subsequently, eyeball tissues collected from treated rats were embedded in optimal cutting temperature compound, sliced into sagittal Sect. (6 μm thick), and frozen sections were prepared for immunofluorescence staining with Anti-vWF (AB7356, Merck Millipore, Germany), sections were then evaluated and imaged with a microscope (Zeiss LSM 880 + Airyscan, Germany). Besides, Total RNA was extracted from neovascularized corneas using Trizol Reagent (15,596,018; Invitrogen), the equal amount of RNA was reverse transcribed to cDNA using a reverse transcription kit (11141es60, Yeasen), quantitative Real-Time PCR (qRT-PCR) was performed with a StepOneTM Real-Time PCR detection system (Applied Biosystems, Alameda, CA, USA) using a SYBR Premix Ex Taq Kit (11201ES08, Yeasen). The primer sequences used to amplify specific gene products are provided in the Table 1. The results of qRT-PCR were analyzed by the comparative CT method and normalized with β-actin as an endogenous reference, and calibrated against the normal control group. Finally, isolated neovascularized corneas were extracted in a cold lysis buffer composed of protease and phosphatase inhibitors, and prepared for Western Blot (WB) assays with primary antibodies for Anti-MMP2 (ab92536, Abcam, Cambridge, UK), Anti-MMP9 (ab76003, Abcam), and Anti-VEGFR2 (ab39638, Abcam). These results were detected by enhanced chemiluminescence reagent (ECL-500; ECL, Lulong Inc., Xiamen, China) and recorded by the transilluminator (ChemiDoc XRS System; Bio-Rad, Philadelphia, PA, USA).

**Table 1:** Primer sequences for the quantitative real-time PCR

Gene	Forward Primer (5’-3’)	Reverse Primer (5’-3’)
*VEGFR2*	CTGCTAGCTGTCGCTCTGTG	CCTCTGTCCCCTGCAAGTAA
*MMP9*	CCTGGAACTCACACAACGTC	GCCTTAGTTCAAGGGCACTG
*MMP2*	GTCTCCCCCAAAACAGACAA	GGTCTCGATGGTGTTCTGGT

### In vivo safety testing of drugs

In order to assess the safety of ocular surface application of eye drops, the normal rats were divided into 5 groups (*n* = 6): PBS, MSCm, NanoSU6668, MNS group and T-MNS (200 µg/mL). The drugs were topically administrated to eyes *via* eye drops at one dosage of 10 µL, with administration occurring twice daily at 9:00 and 21:00. After treated 4 days, all rats were observed under slit lamp microscope, the morphology of cornea and fundus oculi were detected by a small animal optical coherence tomography (OCT) system (Optoprobe, OPIMG, UK) and a small animals fundus imaging system (Optoprobe, OPIMG-L, UK). Meanwhile, the corneal epithelial cells and endothelial cells were examined using a laser-scanning in vivo confocal microscope with a corneal imaging module (HRT3-RCM, Heidelberg Engineering, Germany). Subsequently, the eyeball tissues were promptly extracted upon rat euthanasia and fixed in 4% paraformaldehyde for an overnight period. The corneal tissue section samples were then subjected to hematoxylin-eosin (HE) staining and observed under a light microscope (Eclipse 50i; Nikon, Japan).

### Statistical analyses

Data were analyzed using GraphPad Prism 8.0 software. Statistical analysis was performed by Student’s t-test. The data were presented as means ± standard deviation (SD) unless otherwise indicated. A value of *P* < 0.05 was considered statistically significant.

## Results and discussion

### Preparation and characterization of nanoclusters

**Fig. 1:**
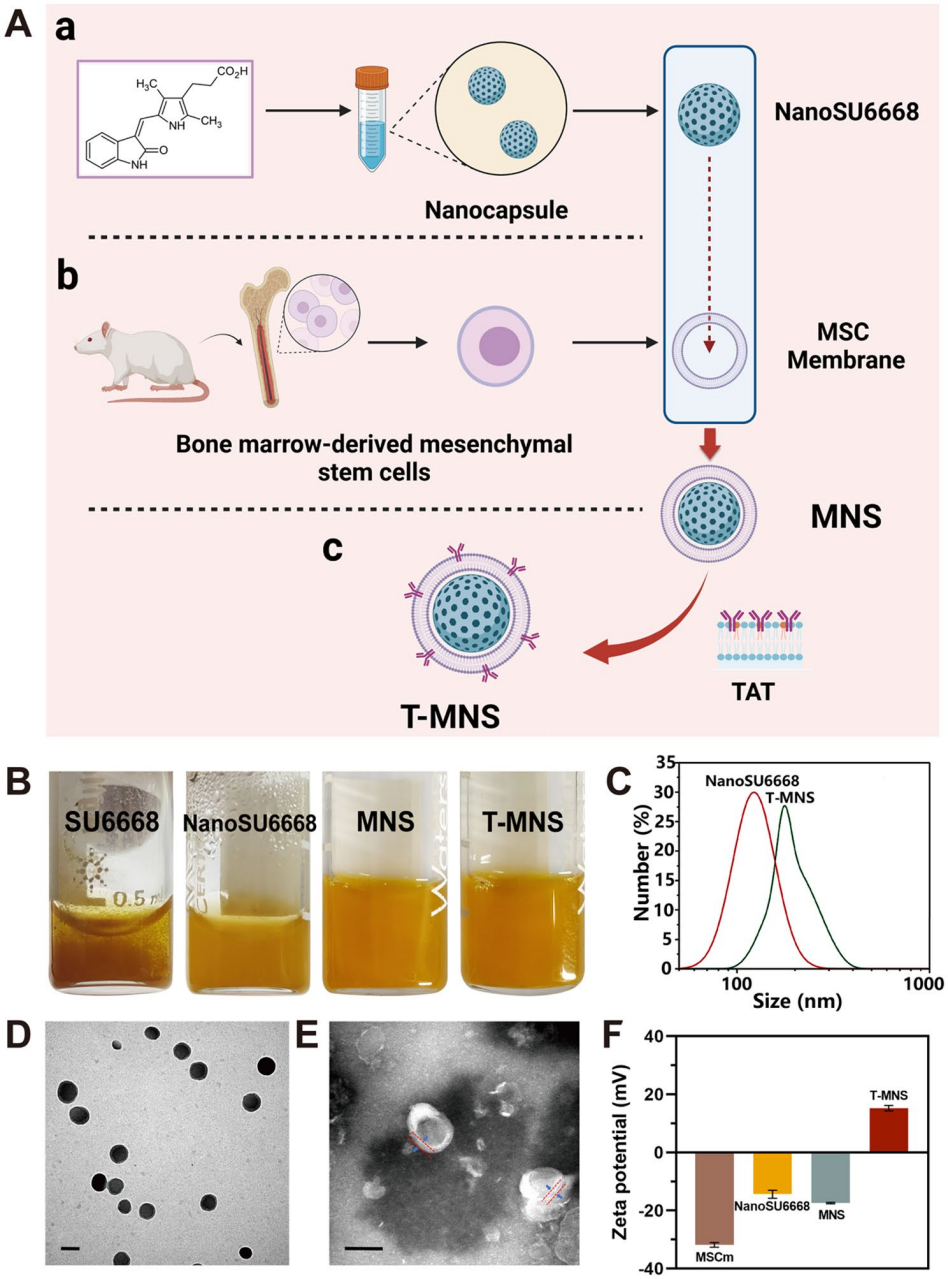
(**A**) Schematic illustration of the preparation of T-MNS system. (**B**) Photo pictures of SU6668, NanoSU6668, MNS and T-MNS. (**C**) DLS of NanoSU6668 and T-MNS. TEM of NanoSU6668 (**D**) and MNS (**E**), (scale bar = 100 nm). (**F**) Zeta-potential of MSCm, NanoSU6668, MNS, and T-MNS

According to our previous study, the NanoSU6668 was prepared by SPFT strategy (Fig. 1A). Flow cytometry analysis showed that isolated MSCs were positive for CD90 (95.6%) (Fig. S1). The hydrophobic drug became aqueous dispersion after the self-assembly reaction (Fig. 1B), and showed long-term dispersion stability at 1 mg/mL of SU6668. Furthermore, the MSCs derived MVs was modified on the surface of NanoSU6668 to improve the biocompatibility, and the further conjugated on the surface of multifunctional nanomedicine. The prepared T-MNS was light yellow dispersed solution, which was uniformly dispersed without precipitation (Fig. 1B). The Dynamic Light Scattering (DLS) study implied that the average particle sizes of NanoSU6668 and T-MNS were 135 and 190 nm, respectively (Fig. 1C). The further TEM image implied that the prepared NanoSU6668 were ultra-small nanoparticles with ideal homodispersity (Fig. 1D). Different from the morphology of pure NPs, TEM image of MNS revealed a spherical core-shell nanostructure with a typical lipid bilayer (Fig. 1E), implying the successful modification of cell membrane on the surface of NanoSU6668 [39]. The fluorescence image of the MVs conjugated with FITC-modified TAT-NHS showed that fluorescence signals accumulate on the surface of MVs (Fig. S2). Furthermore, the zeta-potential of the prepared T-MNS was measured to be 15.23 mV, which different from the electronegative MNS (Fig. 1F), TAT converted negatively charged MNS into positively charged T-MNS, implying TAT was successfully grafted onto the MNS.

### In vitro application of T-MNS treatment

Firstly, the effects of MSCm, NanoSU6668, MNS, and T-MNS on cell cytotoxicity were examined in HCEs and CECs by CCK-8 assay. As shown in Fig. S3, the CCK8 results indicated that drugs exhibited negligible cytotoxicity towards HCEs, even at a high drug concentration of 50 µM. For CECs, T-MNS had a mild cytotoxic effect on CECs only at a concentration of 50 µM. These findings provide compelling evidence that T-MNS exhibited no cytotoxicity to normal cells.

**Fig. 2:**
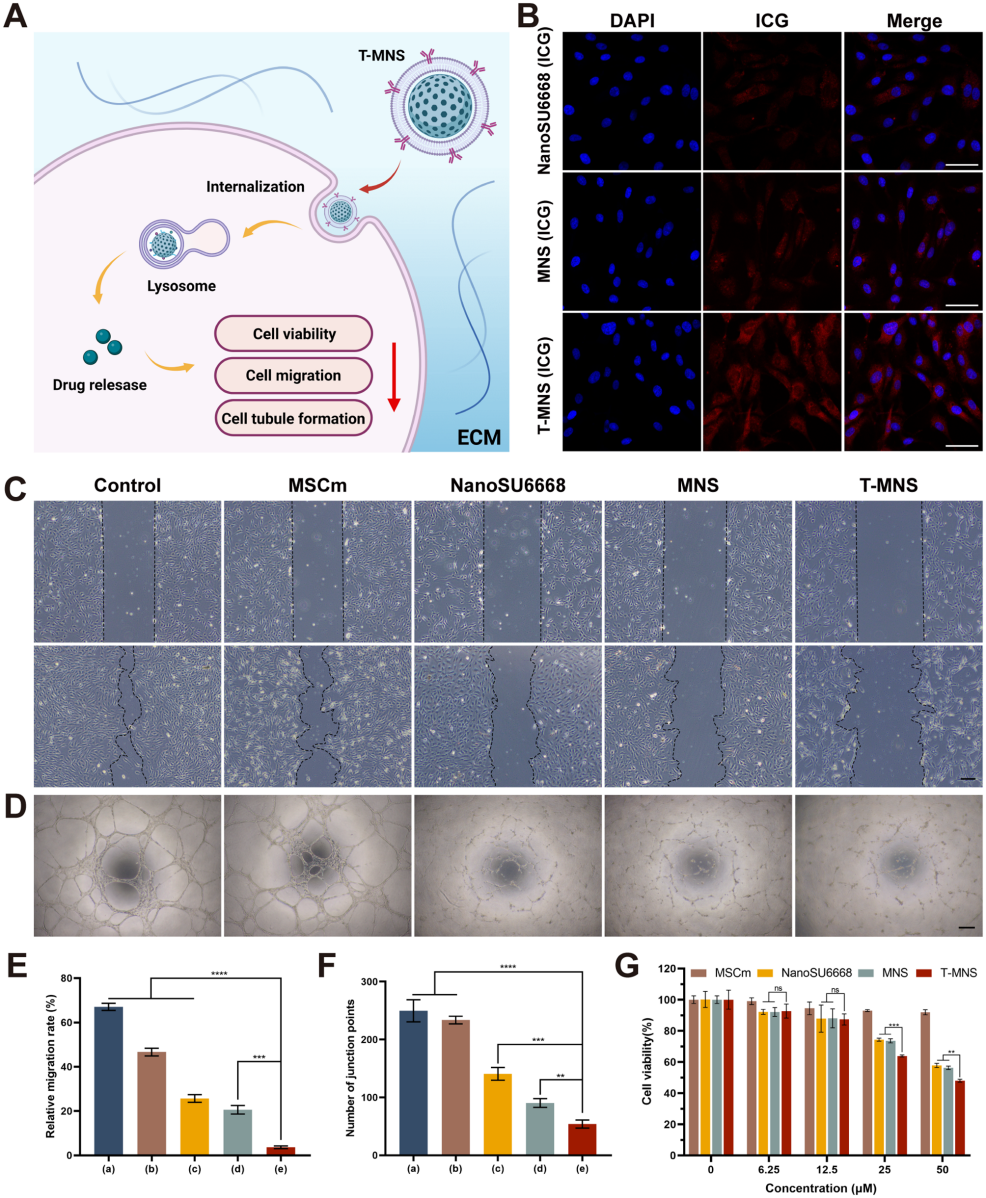
(**A**) Schematic illustration of T-MNS therapeutic strategy for the treatment of HUVECs. (**B**) The cell fluorescence images of the NanoSU6668 (ICG), MNS (ICG) and T-MNS (ICG) incubated with HUVECs, (scale bar = 50 μm). Cells migration images (**C**) and tube formation images (**D**) of HUVECs after different treatments: Control, MSCm, NanoSU6668, MNS and T-MNS, (scale bar = 100 μm). Relative migration rate (**E**) and number of junction points (**F**) of HUVECs after different treatments: Control (**a**), MSCm (**b**), NanoSU6668 (**c**), MNS (**d**) and T-MNS (**e**). (**G**) Cell viability of HUVECs after MSCm, NanoSU6668, MNS and T-MNS treatments with 0, 6.25, 12.5, 25, and 50 µM. Data were presented as means ± SD. *n* = 6 (**B**-**G**), ns: *p* > 0.05, **p < 0.01, ***p < 0.001, ****p < 0.0001

In addition, the HUVECs was applied to study the treatment efficiency of our constructed drug (Fig. 2A). To demonstrate the localization of the drugs within HUVECs, the fluorescence microscopy images were then studied. It was observed that HUVECs treated with T-MNS (ICG) exhibited considerably stronger fluorescent signals compared to those treated with MNS (ICG) and NanoSU6668 (ICG) after a 6 h treatment period (Fig. 2B&S4), in addition, fluorescence microscopy images showed that CECs and HCEs did not endocytosed the T-MNS (ICG) after the same time of treatment (Fig. S5), confirming the targeting effect of T-MNS (ICG) on HUVECs. The targeted effect of T-MNS is crucial for the treatment of CNV, which means that topical administration of T-MNS can achieve the elimination of CNV without damaging normal corneal epithelial and endothelial cells. Cell migration is essential for endothelial cells to form blood vessels during angiogenesis [40]. We then investigated the effect of T-MNS on the migration and angiogenesis of HUVECs. As shown in Fig. 2C&E, when treated with MSCm, NanoSU6668, MNS and T-MNS, the migration rates of HUVECs were reduced. The results implied that the surface-modified MVs also possess therapeutic potential for CNV, consistent with previous research showing that MSCs enhance corneal wound healing and decrease neovascularization [41–43]. Furthermore, it was obvious that T-MNS had the best effect on inhibiting HUVECs migration. Similarly, as shown in Fig. 2D&F, different from the control group that untreated cells formed into tube structure, the cells treated with NanoSU6668, MNS and T-MNS already showed an inhibition of tube formation, and the cells treated with T-MNS exhibited the fewest tube formation. The HUVECs proliferation inhibition of constructed drug were then studied. The findings depicted in Fig. 2G indicate that the MSCm, NanoSU6668, MNS, and T-MNS exhibited a concentration-dependent inhibitory effect on the proliferation of HUVECs, particularly T-MNS, which demonstrated a heightened efficacy in killing HUVECs as the drug concentration increased. These results suggested that T-MNS played an important role in suppressing HUVEC proliferation, migration, and tube formation, largely due to the conversion of negatively charged MNS into positively charged T-MNS by TAT, thereby enhancing the permeability of HUVECs and benefiting for the further in vivo CNV treatment.

### In vivo study of T-MNS for CNV treatment

**Fig. 3:**
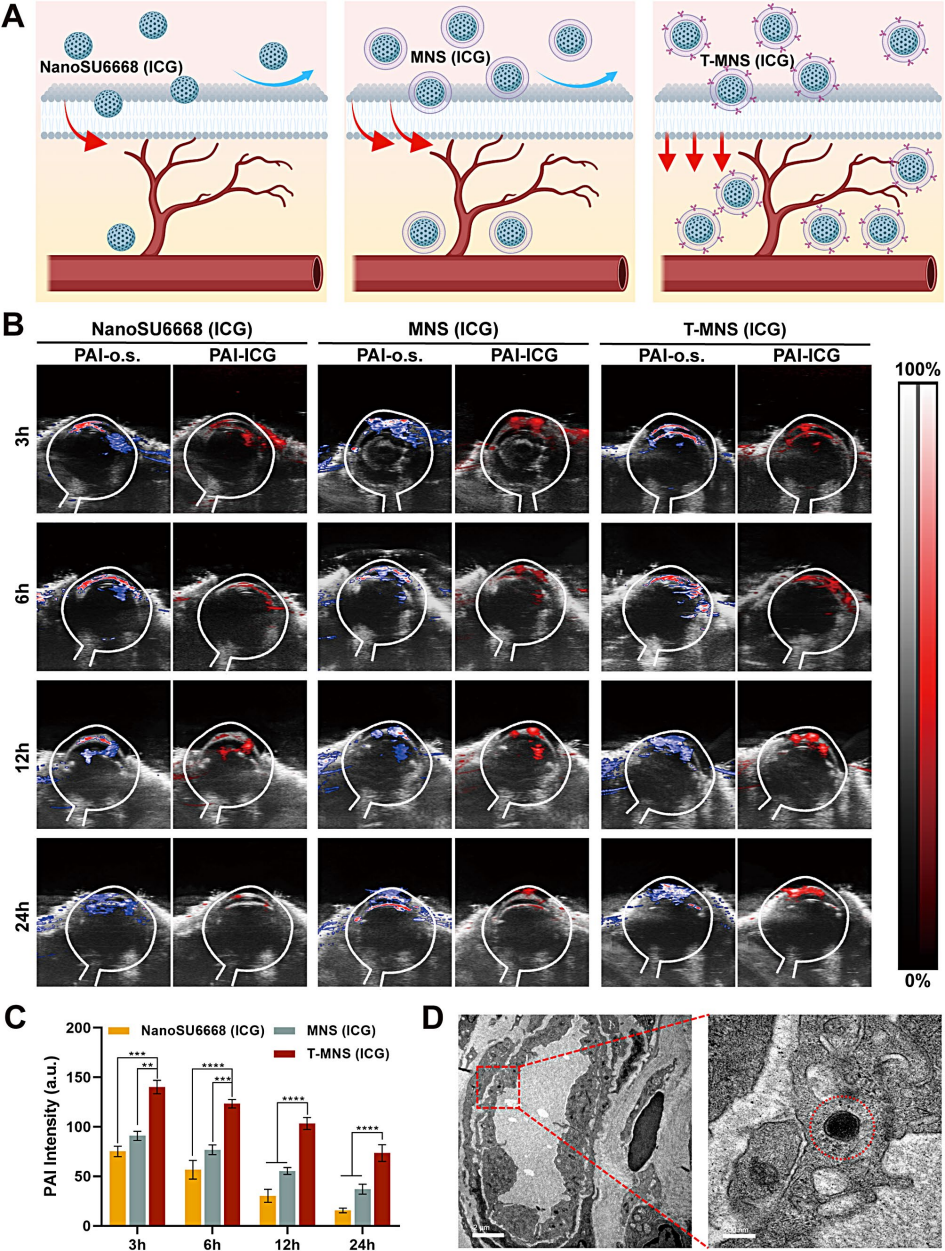
(**A**) Schematic illustration of the drug enrichment and penetration of NanoSU6668 (ICG), MNS (ICG) and T-MNS (ICG). In vivo dual-PAI images of neovascularized eyes (**B**) and PA intensity of cornea (**C**) after topical administration of NanoSU6668 (ICG), MNS (ICG) and T-MNS (ICG). Data were presented as means ± SD. *n* = 6 (**B - C**), **p < 0.01, ***p < 0.001, ****p < 0.0001. (**D**) Thin-section tissue TEM images of cornea after treated with T-MNS (ICG), scale bar = 2 μm (left) or 200 nm (right)

To study the drug accumulation, retention effect of T-MNS in vivo, the CNV rat models were constructed, and the PA-o.s. and PA-ICG dual-imaging system were investigated (Fig. 3A) [44]. As shown in Fig. 3B&C, the PA-o.s. imaging and PA-ICG imaging of corneas matched with each other very well in NanoSU6668 (ICG), MNS (ICG) and T-MNS (ICG) treated groups, implying that PA-ICG signal changes could be used to assess the drug administration effect. The PA intensity of the 3 drugs gradually decreased over time, but it was obvious that the PA intensity in the T-MNS (ICG) treatment group was significantly higher than that in the other groups at all time point. As time goes on, the PA intensity of the T-MNS (ICG) treatment group was about twice that of the MNS (ICG) treatment group after 24 h. Positively charged T-MNS can interact with the negatively charged mucin layer of the tear film, which extends the retention time and absorption on the ocular surface [45, 46]. Furthermore, it was observed that almost all T-MNS (ICG) accumulated at the CNV, with very little drug remained in normal tissues (Fig. 3B). It follows that the T-MNS (ICG) had obvious advantages in targeting CNV, which was suitable for further treatment. Additionally, the tissue TEM of T-MNS (ICG) treated eyeballs were also investigated. As shown in Fig. 3D, the T-MNS (ICG) were found in endothelial cells that formed new blood vessels. It follows that the T-MNS (ICG) had obvious advantages in drug accumulation and retention for CNV in vivo, as well as good penetrability in corneal tissues and a targeting effect toward blood vessels. The above results indicated that T-MNS (ICG) can penetrate the corneal epithelium and accumulate in CNV after 3 h of treatment, and the drug accumulation remained after 24 h. To maintain the effective drug accumulation, it was considered that the optimal medication frequency is twice a day with a 12-hour interval. However, after 3 h of treatment, the accumulation of non-targeted NPs NanoSU6668 (ICG) at CNV was significantly lower than that of T-MNS (ICG), and the accumulation of NanoSU6668 (ICG) was significantly reduced after 12 h treatment. It means that non-targeted modified NPs lack adequate corneal penetration and drug accumulation.

**Fig. 4:**
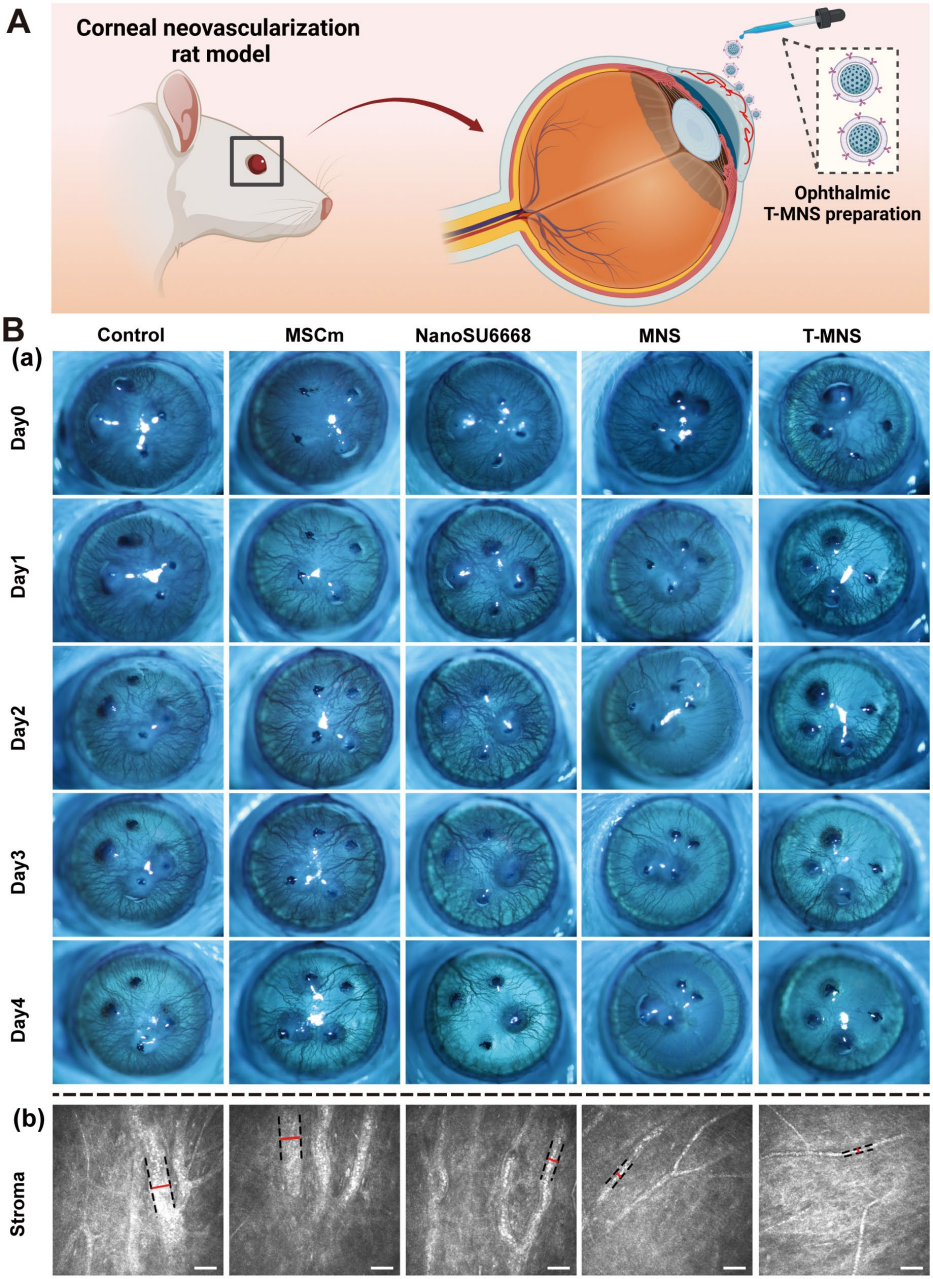
(**A**) Schematic illustration of the T-MNS treatment for CNV rat modeling. (**B**) Slit-lamp images (**a**) and in vivo confocal imaging (**b**) of neovascular eyes before and after PBS, MSCm, NanoSU6668, MNS, T-MNS treatment (scale bar = 50 μm)

To detect the corneal condition during T-MNS treatment for CNV. The CNV rats were treated with eye drops of PBS, MSCm, NanoSU6668, MNS and T-MNS (Fig. 4A). The results showed that after 4 days of treatment, T-MNS at 100 µg/mL showed only a slight therapeutic effect for CNV, and T-MNS at 200 µg/mL showed a noticeable elimination effect on CNV (Fig. S6). Therefore, we selected a drug concentration of 200 µg/mL for dosing in the following experiments. Slit lamp photographic revealed that CNV extends from the limbus to the central cornea following the successful establishment of the CNV model, resulting in corneal edema and cloudiness in all experimental groups. In the NanoSU6668, MNS, and T-MNS treated groups, the inhibition of neovascularization was observed. Notably, the T-MNS treatment exhibited the highest efficacy and most rapid therapeutic effect for CNV among the treated groups, leading to the elimination of almost all large blood vessels and the restoration of cornea transparency within 4 days of treatment (Fig. 4B-a). Quantitative analysis of angiogenesis further revealed that blood vessels markedly diminished after T-MNS treatment, T-MNS showed a significant advantage in eliminating CNV, starting from the second day of treatment and becoming increasingly apparent in subsequent treatments (Fig. S7). Furthermore, in vivo confocal imaging showed that more reduction and thinner of blood vessel in T-MNS treated group than the other treated groups (Fig. 4B-b&S8).

**Fig. 5:**
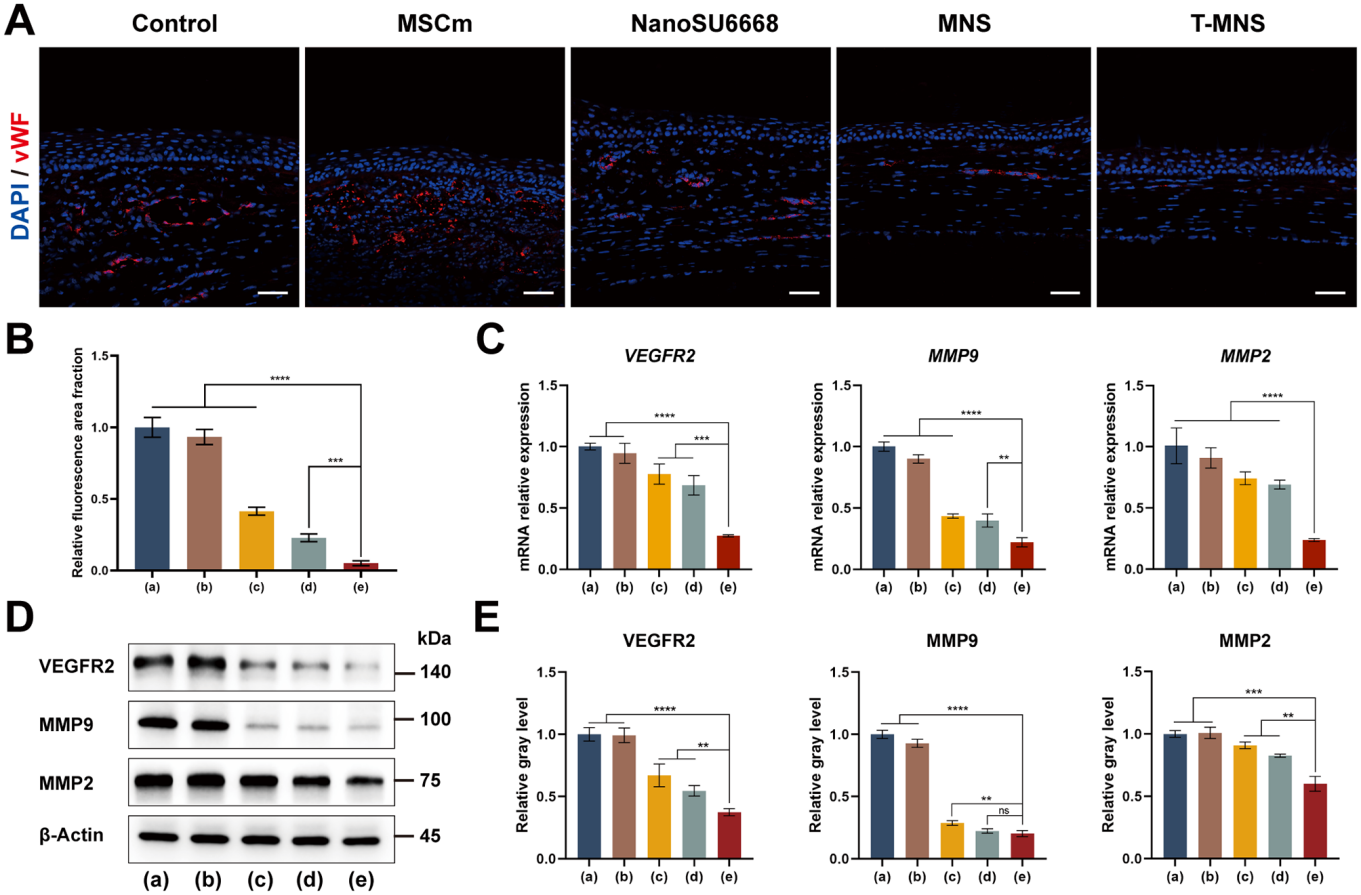
(**A**) The immunofluorescent staining of vWF of corneas after different treatments (scale bar = 50 μm). (**B**) Relative fluorescence area fraction of vWF staining with corneas after PBS (**a**), MSCm (**b**), NanoSU6668 (**c**), MNS (**d**), and T-MNS (**e**) treatments. (**C**) The mRNA relative expression of *VEGFR2*, *MMP9* and *MMP2* in cornea after PBS (**a**), MSCm (**b**), NanoSU6668 (**c**), MNS (**d**), and T-MNS (**e**) treatments. (**D**) WB study of the expression of VEGFR2, MMP9 and MMP2 in cornea after PBS (**a**), MSCm (**b**), NanoSU6668 (**c**), MNS (**d**), and T-MNS (**e**) treatments. (**E**) The gray-scale analysis of WB shows a decrease of VEGFR2, MMP9, and MMP2 expression at the T-MNS treatment group (**e**) compared with PBS (**a**), MSCm (**b**), NanoSU6668 (**c**), and MNS (**d**) treatment group. Data were presented as means ± SD. *n* = 4 (**A - B**), *n* = 6 (**C**), *n* = 3 (**D - E**), **p < 0.01, ***p < 0.001, ****p < 0.0001

Afterwards, the corneas were harvested and studied with von Willebrand factor (vWF), which is routinely used to identify vessels in tissue Sect. [47]. The immunofluorescence staining image showed that the vWF level was decreased after NanoSU6668, MNS, and T-MNS treatments, and the efficiency of T-MNS was apparently higher than other treated groups (Fig. 5A&B). As VEGFR2, MMP-9 and MMP-2 play critical roles in angiogenesis, the expression of VEGFR2, MMP-9 and MMP-2 after different treatments were detected using qRT-PCR and Western Blot Assays [48, 49]. The qRT-PCR revealed that mRNA expression of *VEGFR2*, *MMP-9* and *MMP-2* were significantly decreased in the T-MNS treated (Fig. 5C). As shown in Fig. 5D&E, the protein expression level of VEGFR2, MMP-9 and MMP-2 in the NanoSU6668, MNS and T-MNS treated groups had conspicuous decreases compared with the control group. Furthermore, the inhibitory effect of T-MNS on VEGFR2 and MMP-2 was considerably superior to that of the MNS and NanoSU6668 treatment groups. After treated with MNS and T-MNS, the expression of MMP-9 was reduced to a very low level and benefited for the CNV treatment, regardless that there was no significant difference o between T-MNS and MNS treatment groups. VEGF/VEGFR system is known as a critical regulator of CNV [50], and the VEGFR2 is involved in the main process of mediating VEGF-induced neovascularization [11], which is one of the targets of SU6668. In our study, the T-MNS treatment group showed down-regulation of VEGFR2 gene and protein levels, indicating that the effect of T-MNS on CNV is mainly through inhibiting VEGF/VEGFR signaling pathway. Previous study showed that MMP-2 and MMP-9 prompt CNV by degrading and remolding endothelial basement membrane and extracellular matrix [51]. As a CNV-related inflammation factor, MMP-9 expression in vascularized corneal tissues was positively correlated with VEGF and VEGFR2 expression [52, 53]. Therefore, when VEGFR2 inhibitor was used to block the VEGF/VEGFR2 pathway, the expression of MMP-9 was consistent with VEGFR2, showing a more significant decrease than MMP-2.

**Fig. 6:**
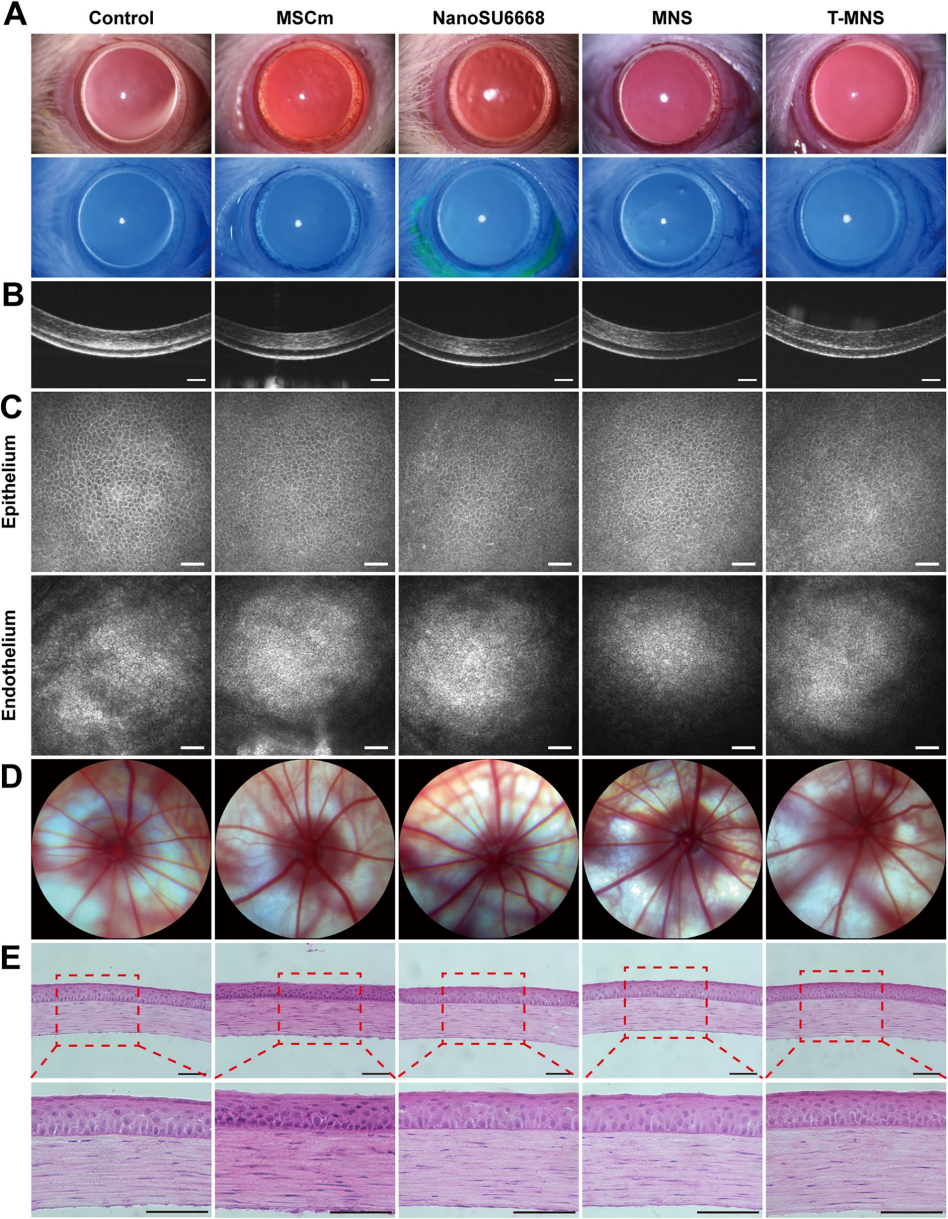
(**A**) Slit-lamp images of normal rat cornea after different treatments. (**B**) OCT images of normal rat cornea after different treatments, (scale bar = 100 μm). (**C**) In vivo confocal imaging of cornea epithelial layer and endothelial layer after different treatments, (scale bar = 50 μm). (**D**) Retinal blood vessel images after different treatments. (**E**) H&E staining images of normal rat cornea after different treatments (scale bar = 100 μm)

In light of the significance of therapeutic safety in the treatment of CNV, additional investigations were undertaken to assess the safety of corneal and retinal blood vessels. Slit lamp images showed normal structure and transparency of cornea in both control and all treatment groups, cornea fluorescein staining images showed corneal epithelium was intact without any defect (Fig. 6A). Corneal thickness and its multilayered structure are important factors of corneal normal morphology, typically assessed through optical coherence tomography (OCT) [54]. The OCT images of corneas in all treatment groups showed a clear multilayered structure with no obvious changes (Fig. 6B). Besides, the utilization of in vivo confocal microscopy facilitates the examination of corneal microstructure, thereby providing a comprehensive and lucid understanding of all corneal layers [55]. As shown in Fig. 6C, the morphology and quantity of corneal epithelial cells and corneal endothelial cells in all treatment groups were basically consistent with those in control group. Additionally, the fundus of the rats was also investigated, fundus photographic revealed there were no damage, hemorrhage or neovascular growth of retinal blood vessels in all treatment groups (Fig. 6D). Furthermore, the H&E staining demonstrated that the cells of corneal epithelium, stroma, and endothelium were arranged regularly with clear multilayer structure, and no organizational changes were found after treatment (Fig. 6E). All these results implied that the prepared T-MNS possessed tissues safety.

### Conclusions

In this study, we prepared a multifunctional T-MNS based eyedrop for CNV treatment through inhibiting angiogenesis-related receptor. Four advantages were summarized for the T-MNS eyedrop: (i) the T-MNS could easily permeate into the corneas, benefit by MSC membrane surface proteins maintained the targeting neovascularization properties and TAT peptide, achieving high drug accumulation in targets and rapid drug clearance in other non-target eye tissues; (ii) the T-MNS treatment effectively and rapidly eliminated neovascularization in CNV, owing to its precise genetic targeting. T-MNS treatment significantly decreased the expression of VEGFR2, MMP-9, and MMP-2, which are crucial genes involved in the process of angiogenesis; (iii) the T-MNS exhibits prolonged retention in the cornea, the low frequency eye drops are patient-friendly and simple to operate; (iv) the T-MNS eye drop with suitable concentration did not cause any damage to other tissues of the eye, including the ocular surface and fundus. In conclusion, the T-MNS eye drop had the potential to treat CNV effectively and safely in a low dosing frequency, which broke new ground for CNV theranostics.

### Electronic supplementary material

Below is the link to the electronic supplementary material.

Supplementary Material 1
