# Supplementary material for: A SU6668 pure nanoparticle-based eyedrops: toward its high drug Accumulation and Long-time treatment for corneal neovascularization
Source: J Nanobiotechnology. 2024 May 27;22:290. doi: 10.1186/s12951-024-02510-8 (PMC11129376; doi:10.1186/s12951-024-02510-8)
Supplement: Supplementary file 1 — Supplementary Material 1 [file 12951_2024_2510_MOESM1_ESM.docx]

**A SU6668 Pure Nanoparticle-based Eyedrops: Toward Its High Drug Accumulation and Long-time Treatment for Corneal Neovascularization**

Han Wu^1,2^^†^, Jinfa Ye^1,2†^, Minjie Zhang^1,4†^, Lingyu Zhang^1,2^, Sijie Lin^1,2^, Qingjian Li^1,2^, Yanbo Liu^1,2^, Yun Han^1,2^, Caihong Huang^1,2^, Yiming Wu^1,2^, Yuhang Cheng^1,2^, Shundong Cai^2^, Lang Ke^1,2^, Gang Liu^3,5*^, Wei Li^1,2*^, Chengchao Chu^1,2,5*^

^1^ *Xiamen University Affiliated Xiamen Eye Center, Eye Institute of Xiamen University, School of Medicine, Xiamen University, Xiamen, 361102, China*

*^2^ Fujian Provincial Key Laboratory of Ophthalmology and Visual Science; Fujian Engineering and Research Center of Eye Regenerative Medicine, Xiamen, 361102, China*

^3^ *State Key Laboratory of Physical Chemistry of Solid Surfaces & The MOE Key Laboratory of Spectrochemical Analysis & Instrumentation, College of Chemistry and Chemical Engineering, Xiamen University, Xiamen, 361002, China*

^4^*Department of Rheumatology and Clinical Immunology, the First Affiliated Hospital of Xiamen University, School of Medicine, Xiamen University, Xiamen, XM, 361000, China, Xiamen Municipal Clinical Research Center for Immune Diseases, Xiamen, XM, 361000, China, Xiamen Key Laboratory of Rheumatology and Clinical Immunology, Xiamen, XM, 361000, China.*

^5^*Shen Zhen Research Institute of Xiamen University, Shenzhen 518057, China.*

* Corresponding authors.

E-mail addresses: gangliu.cmitm@xmu.edu.cn (G. Liu), wei1018@xmu.edu.cn (W. Li), [chuchengchao@xmu.edu.cn](mailto:chuchengchao@xmu.edu.cn) (C. Chu).

^†^ Han Wu, Jinfa Ye and Minjie Zhang contributed equally to this work.


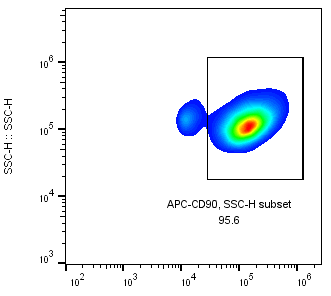


Fig. S1 Flow cytometry analysis for MSCs for surface markers CD90.


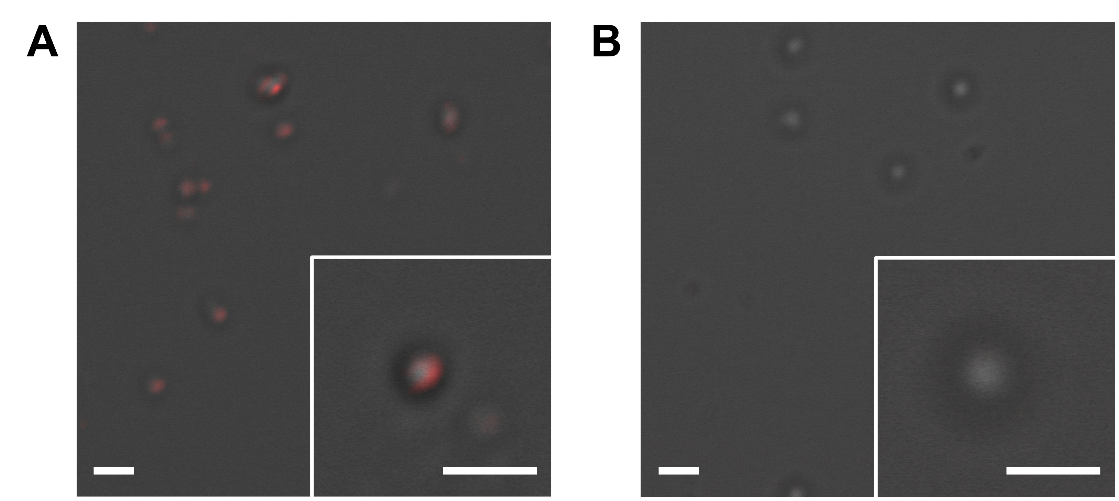


Fig. S2 The fluorescence images of the MVs conjugated with FITC-modified TAT-NHS (A) and the MVs conjugated with TAT-NHS. (scale bar = 1 μm)


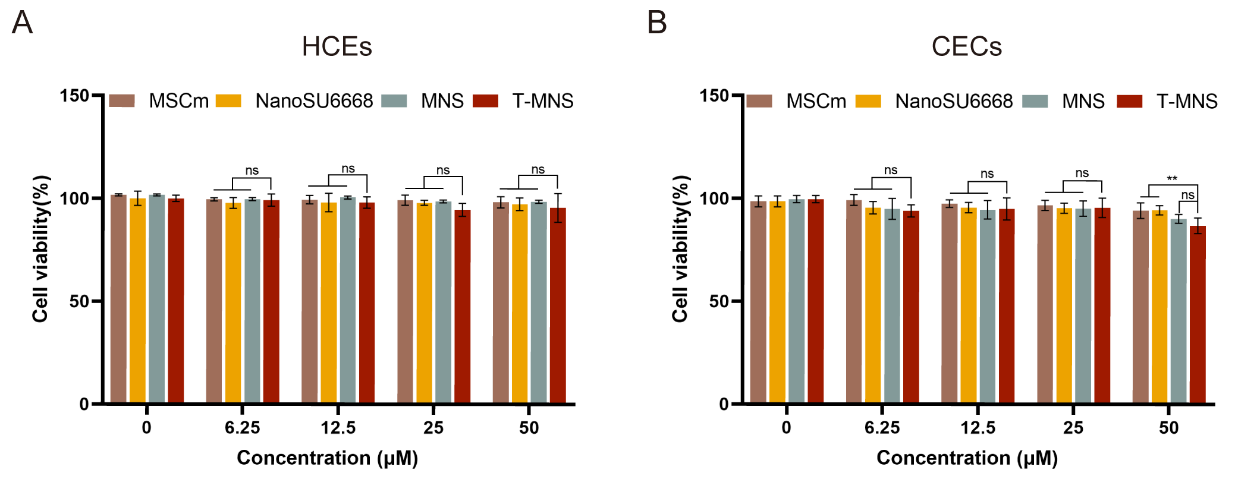


Fig. S3 The CCK-8 studies of MSCm, NanoSU6668, MNS, and T-MNS (6.25 μM, 12.5 μM, 25 μM, 50 μM) in HCEs (A) and CECs (B). Data were presented as means ± SD. n = 6 (A-B), ns: *p* > 0.05, ***p* < 0.01.


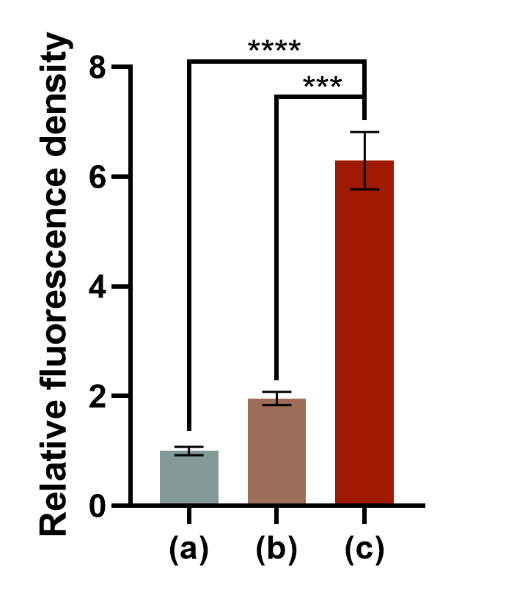


Fig. S4 Relative fluorescence density of the NanoSU6668 (ICG) (a), MNS (ICG) (b) and T-MNS (ICG) (c) incubated with HUVECs. Data were presented as means ± SD. n = 6, ****p* < 0.001, *****p*< 0.0001.


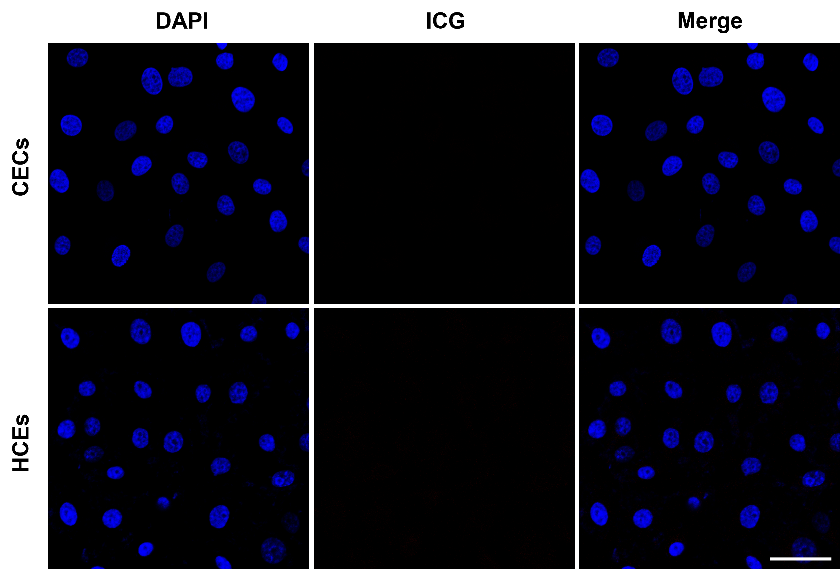


Fig. S5 The cell fluorescence images of the T-MNS (ICG) incubated with CECs and HCEs, (scale bar = 50 μm)


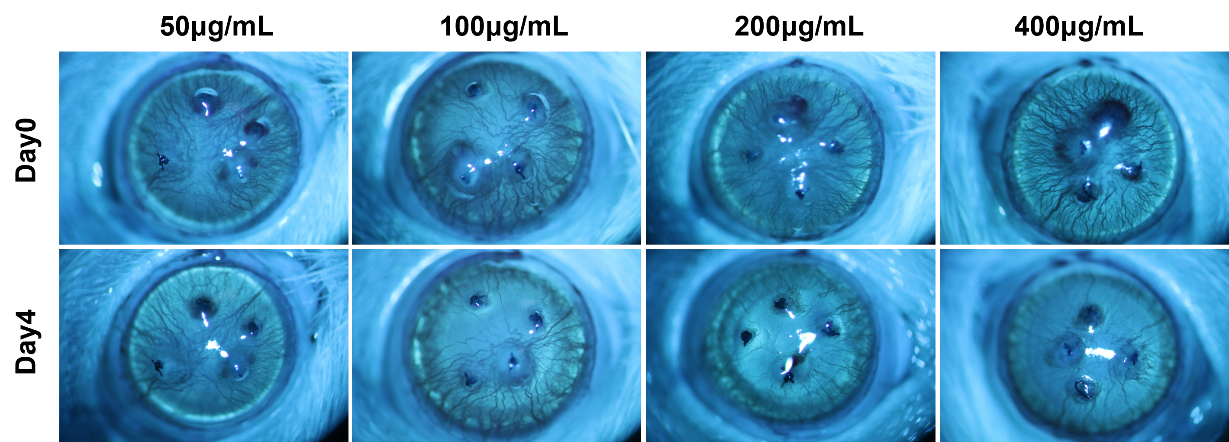


Fig. S6 Slit-lamp images of neovascular eyes before and after T-MNS treatment at a concentration of 50, 100, 200, 400μg/mL.


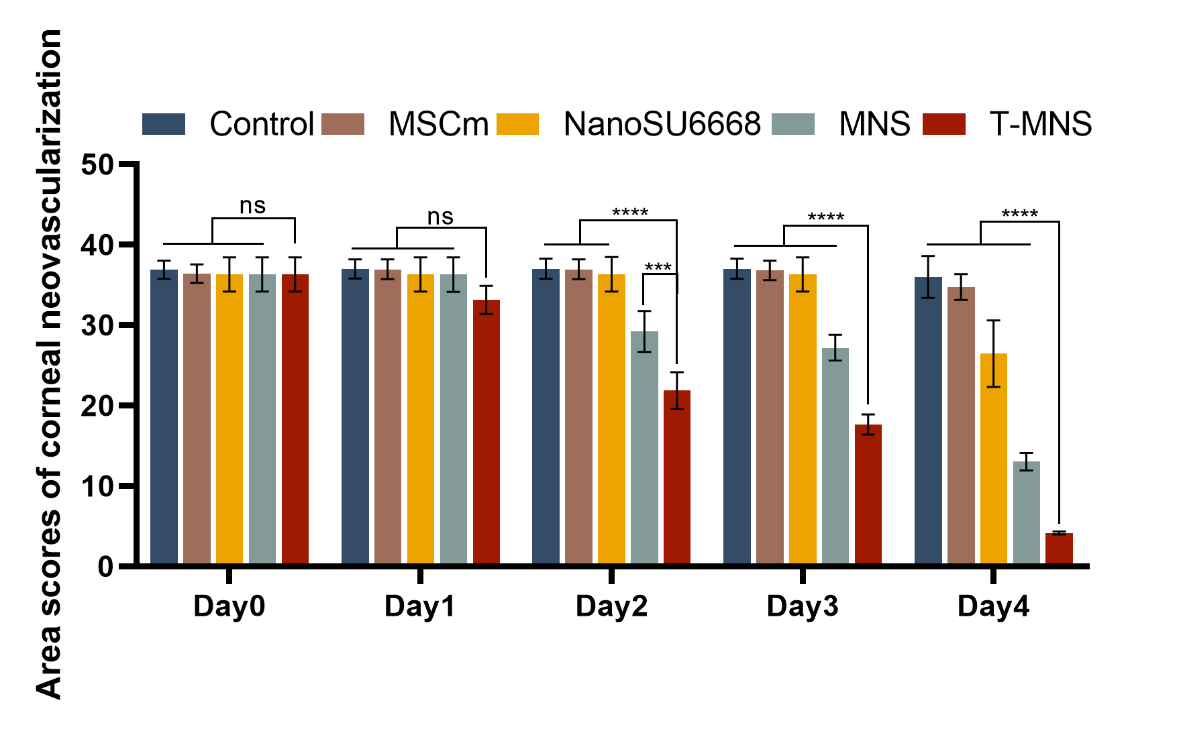


Fig. S7 CNV area scores for each group before and after PBS, MSCm, NanoSU6668, MNS, T-MNS treatment. Data were presented as means ± SD. n = 6, ns: *p* > 0.05, ****p* < 0.001, *****p*< 0.0001.


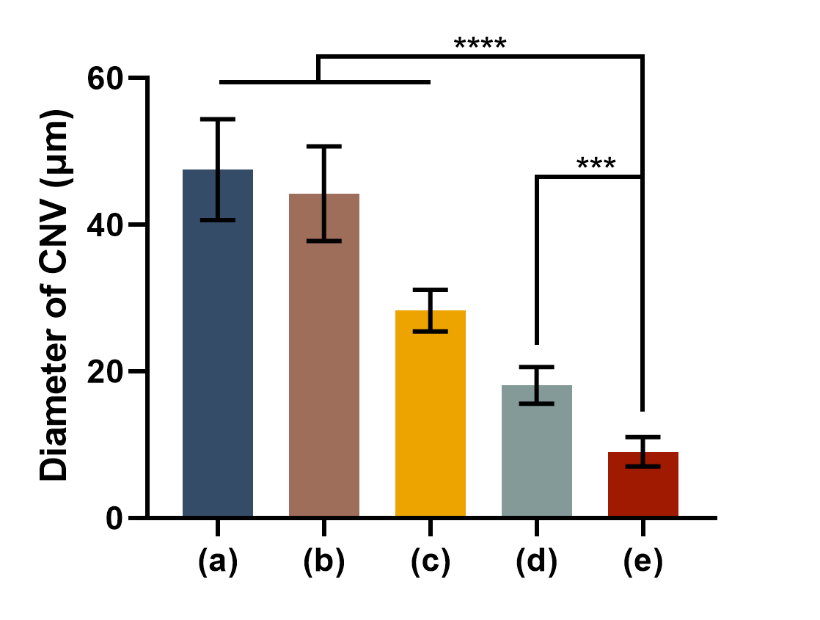


Fig. S8 CNV diameter statistics after PBS (a), MSCm (b), NanoSU6668 (c), MNS (d), T-MNS (e) treatment. Data were presented as means ± SD. n = 5, ***p < 0.001, ****p < 0.0001.
